# The influence of individual characteristics on perceived restorativeness and benefits associated with exposure to nature in a garden

**DOI:** 10.3389/fpsyg.2023.1130915

**Published:** 2023-02-23

**Authors:** Enrico Sella, Chiara Meneghetti, Veronica Muffato, Erika Borella, Elena Carbone, Raffaele Cavalli, Francesca Pazzaglia

**Affiliations:** ^1^Department of General Psychology, University of Padua, Padua, Italy; ^2^Department of Land, Environment, Agriculture, and Forestry, University of Padua, Padua, Italy; ^3^Interuniversity Research Centre in Environmental Psychology (CIRPA), Rome, Italy

**Keywords:** exposure to nature, perceived restorativeness, personality traits, connectedness to nature, affect, memory

## Abstract

This study newly explored the relationship between individual characteristics (i.e., connectedness to nature, a preference for natural rather than built environments, personality, visuospatial preferences) with perceived restorativeness, as well as affective and memory-related benefits of exposure to the nature. Eighty adults were individually exposed to nature by walking in a landscape garden. Measures of connectedness to nature, preference for natural environments, personality traits, and visuospatial preferences were administered. Before and after walking in the garden, participants completed measures of affect (positive and negative emotions) and memory (short-term and working memory, and spatial memory). After walking they completed a Perceived Restorativeness scale. Perceived Restorativeness was found to be significantly explained by Extraversion (personality trait) and Connectedness to Nature. There was no significant influence of individual characteristics on benefits to affect and memory measures. Overall, the results showed that perception of the restorative effect of a natural environment is related to connectedness to nature and personality (extraversion trait). Taken together, our findings highlight the importance of considering individual characteristics to better capture restorative/recovery effects of a natural environment in an individual, and to tailor/implement nature-based solutions to ensure a sustainable urban green environment and to promote quality of life for their citizens.

## 1. Introduction

A natural environment can be perceived as having a restorative potential [see Menardo et al. (2021) for a meta-analysis]. In particular, exposure to nature can have restorative effects on individuals’ positive affect (reducing stress) and improve cognition (i.e., memory and attention), since nature becomes source of fascination, and being in contact with it stimulates the use of involuntary attention. It has indeed been observed that exposure to nature leads to more positive emotions and positive physiological arousal, which reduce perceived stress levels, in the participants exposed to the natural environment when compared with those exposed to the built environment see Yao et al. (2021). These are key aspects of dominant theories regarding why interaction with nature confers such benefits, including: Stress-reduction theory (SRT; Ulrich, 1983), and Attention restoration theory (ART; Kaplan, 1995). Systematic reviews have suggested the –positive – association between long-term exposure to nature (greenspace) and cognition (Ohly et al., 2016; Ricciardi et al., 2022). However, evidence of a beneficial effect of nature exposure are still inconsistent.

Notwithstanding the growing body of evidence on psychological benefits of nature and its restorative potential as a product of a person-environment interaction, little is still known about whether restorativeness and benefits of nature exposure may also depend on individual characteristics.

The existing literature on restorative environments has tended to focus on natural physical/environmental properties (Carrus et al., 2013; Tang et al., 2017), but the restorative effects of nature may also relate to individual attitudes to nature and personal preferences (Berto et al., 2018). Although exposure to natural environments is fundamental for the activation of mechanisms able to restore/replenish directed attention capacity (Kaplan, 1995), and/or recover from stress (Ulrich, 1983), it is also true that some individuals are more prone to appreciate exposure to nature and give restorative effects from it. For instance, the positive effects of nature have been associated with individual’ levels of connectedness to nature–the perceived affective/experiential connection between the self and nature. There is, in fact, evidence of connectedness to nature being associated with its perceived restorativeness (Mayer et al., 2009; Berto and Barbiero, 2017). Differences in connectedness to nature have also been correlated with a preference for natural as opposed to built environments (Nisbet et al., 2009; McMahan and Josh, 2017). Thus, such individual characteristics on connectedness to nature, and environmental preference for natural environments, could be important aspects to account for perceived restorativeness, and–cognitive and affective–benefits associated with nature.

Restorative experiences in a given type of environment can also relate to other individual characteristics, such as general personal dispositions and personality traits, which have been shown to be related to behavior choices, psychological experiences, and well-being (e.g., Roberts et al., 2007). However, there is very few evidence on the associations between personality and perceived restorativeness, and mixed and heterogenous results coming from diverse study settings (laboratory or real environment), and methods (in terms of instruments). Laboratory studies using images/videos found that some personality traits (extroversion–i.e., energetic/outgoing activities-, agreeableness–i.e., the entity/value of being with others-, openness–i.e., the appreciation/curiosity toward art and culture-) correlated with the psychological restorative effect of nature (Felsten, 2014; Senese et al., 2020; Subiza-Pérez et al., 2021), while neuroticism (low emotional stability) was associated with a worse psychological well-being when living near green spaces (Ambrey and Cartlidge, 2017). Surprisingly, only one study assessed personality in relation to perceived restorativeness (Johnsen, 2013). In the study Johnsen (2013), the restorative experience was tested in individuals walking in a natural environment (Norwegian wilderness area), and only conscientiousness (goal-directed and organized thoughts/behaviors) was found to be correlated with restorativeness. Interest in examining the associations between personality and perceived restorativeness and benefits of nature is worthy of consideration to identify which personality traits could explain the source of variability in restorative experiences of people in contact with nature.

People also differ in their ability to acquire and appraise spatial information when interacting with an environment. Visuospatial preferences (or cognitive styles, such as object or spatial preference when handling spatial information) influence how we experience environments (Blazhenkova and Kozhevnikov, 2009) and relate to environment learning accuracy (Pazzaglia and Moè, 2013; Meneghetti et al., 2014). To date, no studies have examined whether and how visuospatial preferences are related to the restorative effects of nature. This issue was newly assessed here.

Overall, restorative experiences in the individual-environment interactions might be influenced not only by environmental properties, but also by individual characteristics, which has received little attention to date. Connectedness to nature and environmental preference, personality traits, and visuospatial preferences, for instance, might contribute in characterizing the restorative value of nature experience, making individuals more (or less) able to perceive the positive effects, and restorative properties of nature. To capture the source of individual variability when individuals interact with nature, whether and to what extent these individual characteristics impact perceived restorativeness and benefits of nature exposure is worth investigating.

The present study thus aimed to examine, for the first time at our knowledge, the relationship between individual characteristics related to connectedness to nature and a preference for natural rather than built environments, personality traits and visuospatial preferences–and the perceived restorativeness of a natural environment (Aim 1). As experiences of real environments are considered more ecologically valid (Berto et al., 2018), our participants walked for around 50 min in a real landscape garden, and its perceived restorativeness was measured immediately afterward. Toward this aim, we hypothesized connectedness to nature and a preference for natural -rather than built- environments to be positively associated with perceived restorativeness (White et al., 2013; Wilkie and Clouston, 2015; McMahan and Josh, 2017; Berto et al., 2018). In line with previous evidence (Johnsen, 2013; Felsten, 2014; Subiza-Pérez et al., 2021), we also expected a positive correlation between perceived restorativeness and extraversion, but not neuroticism (Johnsen, 2013) or psychoticism–i.e., susceptibility to aggressive/impulsive thoughts and behaviors.

Because of the benefits in affect and cognitive aspects like memory (as suggested by ART and SRT), we also examined the relationship between individual characteristics and such benefits (Aim 2) using a questionnaire to assess affect, verbal short-term and working-memory tasks, and a sketch map task to assess spatial memory. Considering previous evidence (Nisbet et al., 2011), we expected both connectedness to nature and a preference for natural rather than built environments to positively correlate with benefits in affect. Only a weak or null association was expected between memory benefits and both perceived restorativeness and affective benefits, as previous evidence found that changes in positive affect after exposure to nature do not correlate with cognitive changes (Schertz and Berman, 2019; Ricciardi et al., 2022). We also expected that affective benefits to positively correlate with personality disposition of extraversion, while limited association with neuroticism or psychoticism might emerged (Johnsen, 2013; Felsten, 2014; Subiza-Pérez et al., 2021). Given the weak and heterogeneous relationships between personality and cognition (Simon et al., 2020), we expected a modest contribution of personality to performance in the cognitive tasks presented here.

Finally, differences in visuospatial preferences with perceived restorativeness and benefits associated with exposure to nature were (newly) investigated, as they have been found related to spatial and navigation abilities in the environment (Pazzaglia and Moè, 2013). We expected object and spatial preferences to be associated more with spatial memory performance (map task), but little or not at all with other (non-spatial) memory tasks (verbal WM task; Meneghetti et al., 2014).

## 2. Materials and methods

### 2.1. Participants

Eighty individuals (58 females) (age range: 21–57; *M* = 31.07; SD = 11.39) volunteered for the study, recruited by word of mouth at the School of Psychology in exchange for course credits. As for all coefficients considered here (see below), a power analysis performed with R (R Core Team, 2019, “pwr” library) showed that 65 people were needed to obtain a sufficient power of 0.80, an effect size of 0.30, and a *p* < 0.05.

All participants were screened using the following inclusion criteria: (a) no depression, with scores under the clinical cut-off of 14 on Beck’s Depression Inventory II (BDI-II; Beck et al., 1996); and (b) no excessive symptoms of anxiety, as measured by the State-Trait Anxiety Inventory Y2 (STAI-Y2; Spielberger, 2010). Participants reported no history of clinically-relevant disorders. We only included participants who were unfamiliar with the study site.

This study was approved by the local University Research Ethics Committee (No. 4254). Participants were informed of the study aims and gave their written informed consent in accordance with the Declaration of Helsinki (World Medical Association, 2013).

### 2.2. Measures

#### 2.2.1. Perceived restorativeness

The Perceived Restorativeness Scale (PRS; Pasini et al., 2014; 11 items) assesses the perceived restorativeness of an environment in terms of: “being away” (PRS-1; 3 items; α = 0.78); “fascination” (PRS-2; 3 items; α = 0.64); “coherence” (PRS-3; 3 items; α = 0.66) (variables of interest). The “scope” subscale was unreliable for the present sample (2 items; α = 0.14), so was not considered in the analyses.

#### 2.2.2. Affect and memory benefits

##### 2.2.2.1. Affect

The Positive and Negative Affective Status (PANAS; Watson et al., 1988; 20 items) measures positive (PANAS-P: α = 0.79) and negative affect (PANAS-N: α = 0.75). Affective benefits were considered in terms of each domain (PANAS-P, PANAS-N), as follow: PANAS “post-test” scores after walking in the garden minus PANAS “pre-test” scores beforehand.

##### 2.2.2.2. Memory

The Forward and Backward Digit Span tasks (FDS and BDS; De Beni et al., 2008) involve participants repeating a series of digits of increasing length in the same or reverse order, respectively. Two parallel versions were created to control the test-retest effect, and the number of correctly recalled series was used as a measure of short-term and working memory performance, respectively. Memory benefits (i.e., FDS and BDS) were derived as follows: scores in the FDS and BDS respectively after walking in the garden minus scores in the FDS and BDS respectively, before walking in the garden.

The Sketch map task consists of naming in writing or drawing as many landmarks as possible in their appropriate positions on a sketch map of the garden (Supplementary Figure 1). Accuracy was assessed in terms of the Number of Missing Landmarks (NML), the SQuare Root of the Canonical Organization (SQRTCO), and the Canonical Accuracy (CA; Gardony et al., 2016).

#### 2.2.3. Individual characteristics questionnaires

##### 2.2.3.1. Connectedness to nature

The Connectedness to Nature Scale (CNS; Mayer and Frantz, 2004; 14 items; adapted and translated for our purposes) assesses to what degree people feel a part of nature (α = 0.88). Higher total score indicates stronger connectedness to nature.

##### 2.2.3.2. Preference for natural rather than built environments

The Preference for Nature Questionnaire (PNQ; McMahan and Josh, 2017; 10 items; adapted and translated for our purposes) assesses individual differences in preferences for natural vs. built environments (α = 0.87). Higher total score indicates the degree of preference for natural environment.

##### 2.2.3.3. Personality

The Eysenck Personality Questionnaire-revised (EPQ-R; Eysenck, 2004; 48-items; Dazzi, 2011) assesses the personality traits. Here we considered Extraversion (12 items; α = 0.64), and Neuroticism (12 items; α = 0.85). Psychoticism (12 items; α = 0.07) was unsuitable for the present sample, so was not considered.

##### 2.2.3.4. Visuospatial preferences

The Object-Spatial Imagery and Verbal Questionnaire (OSIVQ; Blazhenkova and Kozhevnikov, 2009; 45 items) assesses individuals’ preferences for handling spatial information. The visuospatial preferences are indicated by higher scores in the object imagery scale (15 items; α = 0.64), spatial imagery scale (15 items; α = 0.67), and verbal imagery scale (15 items; α = 0.77).

#### 2.2.4. Study site

Villa Revedin Bolasco^1^ is a 19th century complex consisting in the main noble building, the agricultural outbuildings and the garden designed in landscape style, located in Castelfranco Veneto (Italy). The garden is 7.63 hectares in size, with more than 1,000 trees of 65 different species. It includes meadows, ponds, and hills, with a horse-riding arena surrounded by historical statues (Cavallerizza), a Moorish-style greenhouse, and a boathouse (Cavana).

#### 2.2.5. Procedure

Participants signed the consent form and completed the questionnaires in the following order using the Qualtrics platform: CNS, PNQ, EPQ, OSIVQ, STAI-Y2, and BDI-2. One week later, they were involved in individual pre-test and post-test sessions, and in environment exploration in the garden. In the pre-test session, participants completed the PANAS, FDS and BDS. Participants were led to the starting point (Supplementary Figure 1) and invited to explore the garden freely, walking both along gravel paths and elsewhere (e.g., on the meadows). Each participant was granted to a single session of 50-min exposure to the garden. In the post-test session, participants completed the PANAS, FDS and BDS, the PRS-11, and the sketch map task.

## 3. Results

Table 1 shows the correlations between the variables of interest.

**TABLE 1:** Correlations between measures of interest.

	Age	Gender	*E*	*N*	OSIVQ-1	OSIVQ-2	OSIVQ-3	PNQ	CNS	PANAS, P	PANAS, N	FDS	BDS	SQRTCO	NML	CA
Gender ^a^	-0.150	-														
E	0.049	0.050	—													
N	-0.190	0.312**	0.051	—												
OSIVQ-1	0.036	0.125	0.150	0.190	—											
OSIVQ-2	-0.055	-0.575***	-0.172	-0.217	-0.008	—										
OSIVQ-3	-0.060	0.070	0.213	0.094	0.211	-0.118	—									
PNQ	0.114	0.109	0.013	0.051	-0.040	0.101	-0.173	—								
CNS	0.064	0.022	0.188	-0.015	0.184	-0.027	0.148	0.353***	—							
PANAS, P	-0.123	-0.078	-0.002	0.094	0.160	0.077	0.014	0.127	0.100	—						
PANAS, N	-0.005	0.017	0.154	-0.052	-0.094	-0.064	0.113	-0.130	0.167	-0.116	—					
FDS	0.195	-0.073	0.118	-0.029	0.070	-0.065	0.118	-0.133	0.069	-0.071	-0.160	—				
BDS	-0.044	-0.185	-0.059	0.069	-0.128	0.015	-0.200	0.124	0.048	0.255*	0.043	-0.017	—			
NML	-0.042	0.196	-0.008	-0.041	-0.177	-0.220*	0.078	-0.067	-0.189	-0.173	-0.036	-0.121	-0.178	-0.985***	—	
SQRTCO	0.054	-0.164	0.083	0.008	0.240*	0.187	-0.024	-0.001	0.224	0.238*	0.089	0.089	0.191	-0.070	0.228*	—
CA	-0.014	-0.078	0.172	-0.10	-0.081	0.104	0.098	-0.133	0.236	0.062	0.141	0.160	0.261*	0.025	-0.003	-0.063
PRS-1	-0.180	0.261*	0.235*	0.194	0.204	-0.103	0.030	0.237*	0.261*	-0.066	0.034	-0.157	-0.102	-0.003	0.025	-0.068
PRS-2	-0.191	0.047	0.141	-0.065	0.101	-0.052	-0.062	0.223*	0.293**	-0.051	0.076	-0.037	-0.253*	0.033	-0.081	0.183
PRS-3	0.107	0.158	0.118	-0.157	0.134	-0.117	0.063	0.064	0.253*	0.008	0.165	0.009	-0.080	0.195	-0.197	-0.063
PRS, total score	-0.154	0.248*	0.290**	0.069	0.196	-0.199	0.074	0.194	0.348**	-0.008	0.147	-0.063	-0.114	0.106	-0.121	-0.019

OSIVQ-1, object imagery; OSIVQ-2, visual imagery; OSIVQ-3, verbal imagery; E, extroversion; N, neuroticism; PNQ, preference for nature; CNS, connectedness to nature; PANAS, P, positive and negative affective status, positive emotions; PANAS, N, positive and negative affective status, negative emotions; FDS, forward digit span; BDS, backward digit span; NML, number of missing landmarks; SQRTCO, square root of the canonical organization (accuracy considering the NML); CA, canonical accuracy (accuracy not considering the NML); PRS-1, being away; PRS-2, fascination; PRS-3, coherence. ^a^Gender was a dichotomous variable (1 = female, 2 = male). *p < 0.05; **p < 0.01; ***p < 0.001.

See the Supplementary Table 1 for the descriptive statistics.

### 3.1. Aim 1: Individual characteristics and exposure to nature

#### 3.1.1. Correlation analyses

Extraversion was positively associated with the total PRS-11 score and the Being Away domain (*r* = 0.24; *p* = 0.036). There was also a positive correlation between the PNQ and PRS-11 domains of Being Away (*r* = 0.24; *p* = 0.035), and Fascination (*r* = 0.22; *p* = 0.047), indicating that a preference for natural over built environments correlated with more restorative feelings of getting away from daily stress, and enthusiasm for nature. The CNS score correlated positively with the total PRS-11 score and all its subscales (range *r* = 0.25–35).

#### 3.1.2. Regression analyses

Given the exploratory nature of our study, we examined the role of individual characteristics in perceived restorativeness by performing a series of linear models to estimate the overall proportion of variance in perceived restorativeness (PRS-11 total score, and its subscales) explained by the following individual characteristics entered in a unique block: age and gender, as control factors (given their effect on environment experience; Borella et al., 2014; Miola et al., 2021), CNS, PNQ, personality (extraversion, neuroticism), visuospatial preferences (Object, Visual and Verbal imagery, as control factors) (Supplementary material).

**FIGURE 1:**
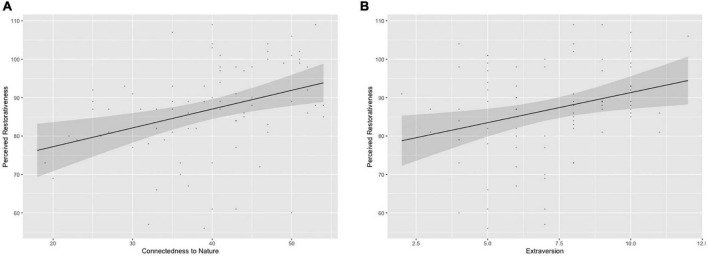
Perceived restorativeness as a function of **(A)** connectedness to nature and **(B)** extraversion.

For the total PRS-11 score, predictors accounted for 28% of the variance. Only extraversion and CNS emerged as significant and positive predictors [β = 0.216, *CI* = (0.01; 2.32), *p* = 0.048; β = 0.249, *CI* = (0.03; 0.67) *p* = 0.034, respectively], indicating that a greater extraversion and connectedness to nature significantly predicted a stronger perception of the restorative effect of walking in a garden (Figure 1). The regression models were run separately for all PRS domains.

For the PRS-1, all the predictors together accounted for 25% of the variance, but no significant predictors emerged (β ranging from −0.187 to 0.195, all *p* < 0.05). For the PRS-2, the predictors accounted for 22% of the variance, with only age emerging as a significant and negative predictor [β = −0.294, *CI* = (−0.07; 0.14), *p* = 0.012]. For the PRS-3, the model was not significant, and no significant predictors emerged (β ranging from −0.230 to 0.221, all *p* < 0.05).

### 3.2. Aim 2: Benefits in affect and memory

#### 3.2.1. Correlation analyses

The benefits in the PANAS-P (positive emotions) correlated positively with the benefits in the BDS (*r* = 0.26; *p* = 0.023) and with higher SQRTCO scores (*r* = 0.24, *p* = 0.037).

The benefits in the BDS correlated negatively with the Fascination domain (*r* = −0.25, *p* = 0.23). The benefits in the BDS were positively associated with the CA score (*r* = 0.26, *p* = 0.022).

Regarding the sketch map task, the object preference correlated positively with a higher SQRTCO (*r* = 0.24, *p* = 0.036), while the spatial preference was negatively associated with the NML scores (*r* = −0.22; *p* = 0.050).

No other significant correlations emerged.

#### 3.2.2. Regression analyses

To further explore the relationship between individual characteristics and benefits in affect and memory, linear models were run, including all benefits to affect (PANAS-P, PANAS-N) and memory (FDS, BDS)^2^, and effects on accuracy in positioning landmarks on the map (NML, SQRTCO, CA) after exposure to nature as dependent variables. No predictors were significant in these models (*R*^2^ ranging from 0.092 to 0.145, all *p* < 0.05) (Supplementary material).

## 4. Discussion

This study newly explored the association between individual characteristics (connectedness to nature, preference for a natural environment, personality, visuospatial preferences) and perceived restorativeness after walking in a garden. Individual differences in explaining benefits to affect and memory, and to spatial learning (sketch map task) were -newly- examined too.

Our results indicated that participants’ connectedness with nature related to their perceived restorativeness and its constitutive domains (in line with Berto et al., 2018), and their preference for nature also correlated with the restorativeness domains of Being away (psychological distance from daily stress) and Fascination (effortless attention to the nature). In other words, people with a greater affinity for natural (over built) environments tended to report restorative experiences of exposure to nature (Aim 1). Our results also indicated an association between personality and perceived restorativeness: in line with the literature, extraversion was associated with perceived restorativeness and its Being Away domain (Johnsen, 2013; Felsten, 2014; Subiza-Pérez et al., 2021), while neuroticism was not (Johnsen, 2013). There was no association between visuospatial preferences and restorativeness, possibly because such preferences are less involved when people interact with restorative experiences of nature than in environment learning processes (Blazhenkova and Kozhevnikov, 2009). Interaction with nature may involve other factors that contribute to the recovery of cognitive resources from attention depletion when in a natural environment (in line with ART). Future studies should try to further clarify this picture.

Our regression analyses confirmed the contribution of some individual characteristics to the restorative effect of nature, with connectedness to nature and extraversion having a major role in explaining overall perceived restorativeness. The restorative effect of nature increases with higher levels of connectedness to nature, and this inclination to feel part of the natural world also seems to reflect how individuals perceive restorative benefits (Berto et al., 2018) when in a natural environment. A greater tendency to focus on positive episodes in individuals higher in extraversion (Anglim et al., 2020) might also lead them to interact actively with the environment, thereby perceiving the restorativeness of nature. In short, the overall restorative effects of nature seem to be caused not only by environmental properties (Ulrich, 1983; Kaplan, 1995), but certain individual characteristics also contribute to how people perceive the restorativeness of nature.

There was no such involvement of individual characteristics in the benefits associated with exposure to nature (Aim 2). The positive effects on affect and memory of individuals interacting with nature seem, at least here, independent of individual differences (as considered here). The benefits to affect and memory revealed different interactions with nature: short-term memory benefits did not correlate with affective changes (Schertz and Berman, 2019), while working memory benefits and spatial memory performance correlated with positive affective changes. Such differences found here might lead to different resources being needed to accomplish memory tasks. Our short-term memory task demanded attentional resources (in line with ART), whereas the WM task and the sketch map task need more active cognitive-control resources to actively process information and, for the visuospatial measure to locate landmarks previously encountered walking in the garden on its layout. Benefits in the latter memory tasks (WM, sketch map task) may also derive from the additional support of positive emotions–as positive affect sustains cognitive/memory-controlled processing (Yang et al., 2013). Regarding the sketch map task, the object imagery score (as measured by OSIVQ-1) was correlated with greater landmark positioning accuracy (as measured by SQRTCO). Individuals who have the preference to form a visual image report greater accuracy in landmark positioning after nature exposure. Furthermore, the object imagery score (as measured by OSIVQ-2) was correlated with fewer missed landmarks (as measured by NML): people who are more prone to form visual image of elements/objects were also those who recall more landmarks (with few missed landmarks) after being in the nature. These results are consistent with previous evidence of an association between sketch map performance accuracy and cognitive processing abilities (such as WM) and attitudes to navigation (e.g., pleasure in exploring; Muffato et al., 2020; see also Meneghetti et al., 2014). A link was also found between benefits to WM and the Fascination domain of restorativeness, suggesting that greater benefits to WM related to effortless attention elicited by exposure to nature. Future research will need to untangle all the processes underlying the benefits associated with exposure to nature.

Some limitations of this study need to be mentioned. Our participants could freely navigate the garden. This can promote the restorative effects of the environment because some people will have gone to the areas they found most attractive/pleasant, but others may have only glanced at (and superficially represented in their mind’s eye) some areas of the garden. Future studies should compare individual differences in restorativeness for people exposed to both natural and built environments (the latter for control purposes) or other activities (viewing images/videos) and different natural environments (urban garden, wilderness). Future studies should also replicate our results including a larger sample size to get robust estimates of the effects of interest. Since definitions of personality can vary across theoretical approaches (Eysenck’s model: Eysenck, 2004; Big Five model: Caprara et al., 1993; HEXACO model: Ashton and Lee, 2007), further studies could extend our results considering other personality dispositions depicted by other useful instruments (such as the Big Five Questionnaire; Caprara et al., 2008). It is also worth noting that restorative environmental properties are typically in function of a given natural environment see Menardo et al. (2021), and our study focused on associations between perceived restorativeness and individual characteristics in people directly exposed to a garden. Within this purpose, the PRS-11 (Pasini et al., 2014) is considered as a valid tool for measuring restorativeness when individuals are -still- exposed, or right after being exposed, to a natural environment (like the garden used here), rather than prior to exposure to a natural environment. Nonetheless, to confirm the present results and to better capture the role of perceived restorativeness, future studies should include other self-report (Berto et al., 2018) or objective/behavioral measures (Lin et al., 2014) which assess restorativeness levels both before and after nature exposure.

Noteworthy, we attempted to account individual differences for restorativeness and benefits after a single 50-min session of nature exposure. However, the time of exposure to nature is still an aspect debated in the literature (Barton and Pretty, 2010). Systematic reviews have suggested that laboratory studies reported benefits in stress level and cognition after a few seconds or minutes of exposure to nature (Yao et al., 2021). Field studies, in contrast, adopted both short-term (range of time: 10–210 min, Yao et al., 2021; 10–90 min; Mason et al., 2021) and long-term exposure (i.e., months, years) by visual access or frequency to greenspace (de Keijzer et al., 2016; Ricciardi et al., 2022) and find benefits. Thus, future studies should deepen the role of exposure to nature. It is also plausible that, alongside the basic psychological mechanisms of restorativeness (in line with ART) and/or through the physiological deactivation (in line with SRT), other individual factors might interact with the time of exposure, such as being engaged with natural activities (e.g., camping, sport exercises) or the familiarity with a given natural environment (Ricciardi et al., 2022). Further, as our participants were unfamiliar with the garden of Villa Revedin Bolasco, it would be interesting to clarify the contribution of both frequency and familiarity with this “restorative” garden. These variables will need to be into account in future studies. Finally, considering the cross-sectional nature of the present study, a follow-up assessment to examine the role of individual characteristics and differences over long-term exposure to nature merits to be assessed.

In conclusion, this study showed that also individual characteristics should be considered when examining the perceived psychologically restorative effect of being in a natural environment. After walking in a garden, the perceived restorativeness correlates with being more extravert and connected to nature. The benefits of nature to some aspects of memory (i.e., working memory) and affect (positive emotions) are also associated with how well the environment is recalled.

## Data availability statement

The data that support the conclusions of this article will be made available by the corresponding author upon reasonable request. Requests to access this dataset should be directed to ES, enrico.sella@unipd.it.

## Ethics statement

The studies involving human participants were reviewed and approved by the University of Padova, Research Ethics Committee (No. 4254). The patients/participants provided their written informed consent to participate in this study.

## Author contributions

All authors listed have made a substantial, direct, and intellectual contribution to the work, and approved it for publication.

## Footnotes

1
https://goo.gl/maps/FBnpobK59uzE7WVe9

2 A series of repeated ANOVAs were run separately on participants’ pre-exposure and post-exposure affective and cognitive measures. Regarding affect, the results showed a significant main effect of time, with improvement from the pre to post-test only in reducing negative emotions [PANAS-N; F(1,79) = 51.164, *p* < 0.001, *n^2^p* = 0.393], while for positive emotions it was not significant [F(1,79) = 0.228, *p* = 0.634, *n^2^p* = 0.003]. Regarding memory, the significant main effect of time, with improvement from pre- to post-test, emerged for short-term memory [FDS; F(1,79) = 9.968, *p* = 0.002, *n^2^p* = 0.112], but not for working memory [BDS; F(1,79) = 2.578, *p* = 0.112, *n^2^p* = 0.032]. Descriptive statistics are provided in the Supplementary material.

## References

[B1] Ambrey C. L. Cartlidge N. (2017). Do the psychological benefits of greenspace depend on one’s personality? *Pers. Individ. Differ.* 116 233–239. 10.1016/j.paid.2017.05.001

[B2] Anglim J. Horwood S. Smillie L. D. Marrero R. J. Wood J. K. (2020). Predicting psychological and subjective well-being from personality: A meta-analysis. *Psychol. Bull.* 146 279–323. 10.1037/bul0000226 31944795

[B3] Ashton M. C. Lee K. (2007). Empirical, theoretical, and practical advantages of the HEXACO model of personality structure. *Pers. Soc. Psychol. Rev.* 11 150–166. 10.1177/1088868306294907 18453460

[B4] Barton J. Pretty J. (2010). What is the best dose of nature and green exercise for improving mental health? A multi-study analysis. *Environ. Sci. Technol.* 44 3947–3955. 10.1021/es903183r 20337470

[B5] Beck A. T. Steer R. A. Ball R. Ranieri W. F. (1996). Comparison of beck depression inventories-IA and-II in psychiatric outpatients. *J. Pers. Assess.* 67 588–597. 10.1207/s15327752jpa6703_13 8991972

[B6] Berto R. Barbiero G. (2017). How the psychological benefits associated with exposure to nature can affect pro-environmental behavior. *Ann. Cogn. Sci.* 1 16–20. 10.3389/fpsyg.2018.02344 30574105 PMC6292239

[B7] Berto R. Barbiero G. Barbiero P. Senes G. (2018). An individual’s connection to nature can affect perceived restorativeness of natural environments. Some observations about biophilia. *Behav. Sci.* 8:34. 10.3390/bs8030034 29510581 PMC5867487

[B8] Blazhenkova O. Kozhevnikov M. (2009). The new object-spatial-verbal cognitive style model: Theory and measurement. *Appl. Cogn. Psychol.* 23 638–663. 10.1002/acp.1473

[B9] Borella E. Meneghetti C. Ronconi L. De Beni R. (2014). Spatial abilities across the adult life span. *Dev. Psychol.* 50 384–392. 10.1037/a0033818 23895173

[B10] Caprara G. V. Barbaranelli C. Borgogni L. Perugini M. (1993). The “Big Five Questionnaire”: A new questionnaire to assess the five factor model. *Pers. Individ. Differ.* 15 281–288. 10.1016/0191-8869(93)90218-R

[B11] Caprara G. V. Barbaranelli C. Borgogni L. Vecchione M. (2008). *BFQ-2. Big five questionnaire 2.* Firenze: Giunti Organizzazioni Speciali.

[B12] Carrus G. Lafortezza R. Colangelo G. Dentamaro I. Scopelliti M. Sanesi G. (2013). Relations between naturalness and perceived restorativeness of different urban green spaces. *Psyecology* 4 227–244. 10.1174/217119713807749869 25131292

[B13] Dazzi C. (2011). The eysenck personality questionnaire–revised (EPQ-R): A confirmation of the factorial structure in the Italian context. *Pers. Individ. Differ.* 50 790–794. 10.1016/j.paid.2010.12.032

[B14] De Beni R. Borella E. Carretti B. Marigo C. Nava L. A. (2008). *BAC. Portfolio per la valutazione del benessere e delle abilità cognitive nell’età adulta e avanzata [The assesment of well-being and cognitive abilities in adulthood and aging].* Firenze: Giunti OS.

[B15] de Keijzer C. Gascon M. Nieuwenhuijsen M. J. Dadvand P. (2016). Long-term green space exposure and cognition across the life course: A systematic review. *Curr. Environ. Health Rep.* 3 468–477. 10.1007/s40572-016-0116-x 27730509

[B16] Eysenck M. W. (2004). *Psychology: An international perspective.* Oxfordshire: Taylor and Francis.

[B17] Felsten G. (2014). Personality predicts perceived potential for attention restoration of natural and urban scenes/La personalidad predice el potencial percibido de restauración atencional de los paisajes naturales y urbanos. *Psyecology* 5 37–57. 10.1080/21711976.2014.881663

[B18] Gardony A. L. Taylor H. A. Brunyé T. T. (2016). Gardony map drawing analyzer: Software for quantitative analysis of sketch maps. *Behav. Res. Methods* 48 151–177. 10.3758/s13428-014-0556-x 25673320

[B19] Johnsen S. ÅK. (2013). Exploring the use of nature for emotion regulation: Associations with personality, perceived stress, and restorative outcomes. *Nord. Psychol.* 65 306–321. 10.1080/19012276.2013.851445

[B20] Kaplan S. (1995). The restorative benefits of nature: Toward an integrative framework. *J. Environ. Psychol.* 15 169–182. 10.1016/0272-4944(95)90001-2

[B21] Lin Y. H. Tsai C. C. Sullivan W. C. Chang P. J. Chang C. Y. (2014). Does awareness effect the restorative function and perception of street trees? *Front. Psychol.* 5:906. 10.3389/fpsyg.2014.00906 25177309 PMC4133958

[B22] Mason L. Ronconi A. Scrimin S. Pazzaglia F. (2021). Short-term exposure to nature and benefits for students’ cognitive performance: A review. *Educ. Psychol. Rev.* 34 609–647. 10.1007/s10648-021-09631-8

[B23] Mayer F. S. Frantz C. M. (2004). The connectedness to nature scale: A measure of individuals’ feeling in community with nature. *J. Environ. Psychol.* 24 504–515. 10.1016/j.jenvp.2004.10.001

[B24] Mayer F. S. Frantz C. M. Bruehlman-Senecal E. Dolliver K. (2009). Why is nature beneficial? The role of connectedness to nature. *Environ. Behav.* 41 607–643. 10.1177/0013916508319745

[B25] McMahan E. A. Josh P. (2017). Measuring preference for natural versus built environments: Initial validation of the preference for nature questionnaire. *Ecopsychology* 9 161–171. 10.1089/eco.2017.0009

[B26] Menardo E. Brondino M. Hall R. Pasini M. (2021). Restorativeness in natural and urban environments: A meta-analysis. *Psychol. Rep.* 124 417–437. 10.1177/0033294119884063 31694463

[B27] Meneghetti C. Labate E. Grassano M. Ronconi L. Pazzaglia F. (2014). The role of visuospatial and verbal abilities, styles and strategies in predicting visuospatial description accuracy. *Learn. Individ. Differ.* 36 117–123. 10.1016/j.lindif.2014.10.019

[B28] Miola L. Meneghetti C. Toffalini E. Pazzaglia F. (2021). Environmental learning in a virtual environment: Do gender, spatial self-efficacy, and visuospatial abilities matter? *J. Environ. Psychol.* 78:101704. 10.1016/j.jenvp.2021.101704

[B29] Muffato V. Meneghetti C. De Beni R. (2020). The role of visuo-spatial abilities in environment learning from maps and navigation over the adult lifespan. *Br. J. Psychol.* 111 70–91. 10.1111/bjop.12384 30927263

[B30] Nisbet E. K. Zelenski J. M. Murphy S. A. (2009). The nature relatedness scale: Linking individuals’ connection with nature to environmental concern and behavior. *Environ. Behav.* 41 715–740. 10.1177/0013916508318748

[B31] Nisbet E. K. Zelenski J. M. Murphy S. A. (2011). Happiness is in our nature: Exploring nature relatedness as a contributor to subjective well-being. *J. Happiness. Stud*. 12, 303–322. 10.1007/s10902-010-9197-7

[B32] Ohly H. White M. P. Wheeler B. W. Bethel A. Ukoumunne O. C. Nikolaou V. (2016). Attention restoration theory: A systematic review of the attention restoration potential of exposure to natural environments. *J. Toxicol. Environ. Health. B. Crit. Rev.* 19, 305–343. 10.1080/10937404.2016.1196155 27668460

[B33] Pasini M. Berto R. Brondino M. Hall R. Ortner C. (2014). How to measure the restorative quality of environments: The PRS-11. *Proc. Soc.* 159 293–297. 10.1016/j.sbspro.2014.12.375

[B34] Pazzaglia F. Moè A. (2013). Cognitive styles and mental rotation ability in map learning. *Cogn. Process.* 14 391–399. 10.1007/s10339-013-0572-2 23771207

[B35] R Core Team (2019). *R: A language and environment for statistical computing.* Vienna: R Foundation for Statistical Computing.

[B36] Ricciardi E. Spano G. Lopez A. Tinella L. Clemente C. Elia G. (2022). Long-term exposure to greenspace and cognitive function during the Lifespan: A systematic review. *Int. J. Environ. Res. Public Health* 19:11700. 10.3390/ijerph191811700 36141977 PMC9517665

[B37] Roberts B. W. Kuncel N. R. Shiner R. Caspi A. Goldberg L. R. (2007). The power of personality: The comparative validity of personality traits, socioeconomic status, and cognitive ability for predicting important life outcomes. *Perspect. Psychol. Sci.* 2 313–345. 10.1111/j.1745-6916.2007.0004 26151971 PMC4499872

[B38] Schertz K. E. Berman M. G. (2019). Understanding nature and its cognitive benefits. *Curr. Dir. Psychol. Sci.* 28 496–502. 10.1177/0963721419854100

[B39] Senese V. P. Pascale A. Maffei L. Cioffi F. Sergi I. Gnisci A. (2020). “The influence of personality traits on the measure of restorativeness in an urban park: a multisensory immersive virtual reality study,” in *Neural approaches to dynamics of signal exchanges*, ed Springer (Singapore: Springer), 347–357.

[B40] Simon S. S. Lee S. Stern Y. (2020). Personality-cognition associations across the adult life span and potential moderators: Results from two cohorts. *J. Pers.* 88 1025–1039. 10.1111/jopy.12548 32199032 PMC7484019

[B41] Spielberger C. D. (2010). *State-trait anxiety inventory. The Corsini encyclopedia of psychology*. Hoboken: Wiley, 1.

[B42] Subiza-Pérez M. Pasanen T. Ratcliffe E. Lee K. Bornioli A. de Bloom J. (2021). Exploring psychological restoration in favorite indoor and outdoor Urban places using a top-down perspective. *J. Environ. Psychol.* 78:101706. 10.1016/j.jenvp.2021.101706

[B43] Tang I. C. Tsai Y. P. Lin Y. J. Chen J. H. Hsieh C. H. Hung S. H. (2017). Using functional Magnetic Resonance Imaging (fMRI) to analyze brain region activity when viewing landscapes. *Landsc. Urban Plan.* 162 137–144. 10.1016/j.landurbplan.2017.02.007

[B44] Ulrich R. S. (1983). “Aesthetic and affective response to natural environment,” in *Behavior and the natural environment*, eds Altman I. Wohlwill J. F. (Boston, MA: Springer), 85–125.

[B45] Watson D. Clark L. A. Tellegen A. (1988). Development and validation of brief measures of positive and negative affect: The PANAS scales. *J Pers Soc Psychol* 54 1063–1070. 3397865 10.1037//0022-3514.54.6.1063

[B46] White M. P. Pahl S. Ashbullby K. Herbert S. Depledge M. H. (2013). Feelings of restoration from recent nature visits. *J. Environ. Psychol.* 35 40–51. 10.1016/j.jenvp.2013.04.002

[B47] Wilkie S. Clouston L. (2015). Environment preference and environment type congruence: Effects on perceived restoration potential and restoration outcomes. *Urban Urban Green* 14 368–376. 10.1016/j.ufug.2015.03.002

[B48] World Medical Association (2013). World Medical Association Declaration of Helsinki: Ethical principles for medical research involving human subjects. *JAMA* 310 2191–2194. 10.1001/jama.2013.281053 24141714

[B49] Yang H. Yang S. Isen A. M. (2013). Positive affect improves working memory: Implications for controlled cognitive processing. *Cogn. Emot.* 27 474–482. 22917664 10.1080/02699931.2012.713325

[B50] Yao W. Zhang X. Gong Q. (2021). The effect of exposure to the natural environment on stress reduction: A meta-analysis. *Urban Urban Green* 57:126932.

