# Supplementary material for: The influence of individual characteristics on perceived restorativeness and benefits associated with exposure to nature in a garden
Source: Front Psychol. 2023 Feb 23;14:1130915. doi: 10.3389/fpsyg.2023.1130915 (PMC9995947; doi:10.3389/fpsyg.2023.1130915)
Supplement: Supplementary file 1 [file Table_1.docx]

Supplementary Material

The Influence of Individual Characteristics on Perceived Restorativeness and Benefits associated with Exposure to Nature in a Garden

**Enrico Sella^1^*, Chiara Meneghetti^1^, Veronica Muffato^1^, Erika Borella^1^, Elena Carbone^1^, Raffaele Cavalli^2^, Francesca Pazzaglia^1,3^**

^1^Department of General Psychology, University of Padova, Italy

^2^Department of Land, Environment, Agriculture and Forestry, University of Padova, Italy

^3^Interuniversity Research Centre in Environmental Psychology (CIRPA), Rome, Italy

*** Correspondence:**Enrico Sella
[enrico.sella@unipd.it](mailto:enrico.sella@unipd.it)

# Supplementary Figures

**Figure S1.** Panel A: Villa Revedin Bolasco on Google Map with landmarks positions. Panel B: the sketch map


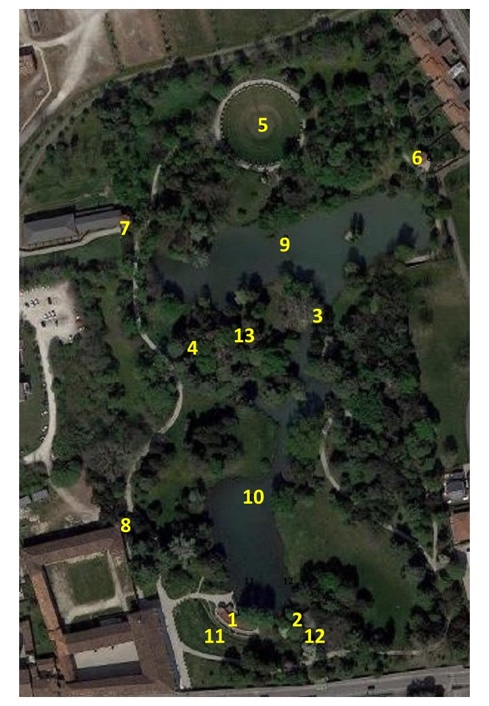

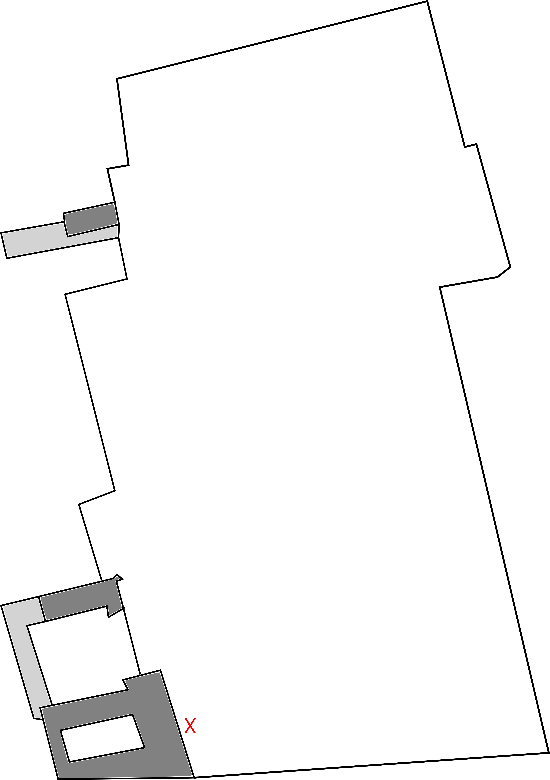


# Supplementary Tables

# Table S1. Landmarks identified within the garden

| **#** | **Landmarks** |
| --- | --- |
| 1 | Greenhouse |
| 2 | *Cavana* |
| 3 | East bridge |
| 4 | West bridge |
| 5 | *Cavallerizza* |
| 6 | East dovecote |
| 7 | West dovecote |
| 8 | Toilette |
| 9 | North lake |
| 10 | South lake |
| 11 | Lake near the greenhouse |
| 12 | Lake behind the *cavana* |
| 13 | Island |

**Table S2.** Characteristics of the Participants

| N=80 | ***M*** | ***SD*** |
| --- | --- | --- |
| Age | 31.34 | 11.56 |
| Female (%) | 73% | - |
| Education | 16.69 | 3.39 |
| STAI-Y2 | 27.65 | 9.31 |
| BDI-2 | 3.59 | 3.30 |
| *Individual characteristics* |  |  |
| PNQ | 49.61 | 9.91 |
| CNS | 39.63 | 8.74 |
| QSIVQ-1 | 40.79 | 5.95 |
| QSIVQ-2 | 43.01 | 12.87 |
| QSIVQ-3 | 42.96 | 9.80 |
| E | 7.14 | 2.28 |
| N | 5.10 | 3.27 |
| *Benefits associated with nature* |  |  |
| *Affective status* |  |  |
| **^**PANAS, P | .34 | 6.32 |
| **^**PANAS, N | -2.38 | 2.97 |
| *Memory* |  |  |
| **^**FDS | .28 | .19 |
| **^**BDS | .19 | 1.05 |
| *Sketch map task* |  |  |
| NML | 7.46 | 2.04 |
| SQRTCO | .38 | .14 |
| CA | .82 | .12 |
| *Perceived Restorativeness* |  |  |
| PRS-1 | 25.60 | 5.10 |
| PRS-2 | 26.15 | 3.17 |
| PRS-3 | 19.96 | 5.19 |
| PRS, total | 86.84 | 12.29 |

*Note.* STAI-Y2= State Trait Anxiety, Y2; BDI-2=Beck Depression Inventory, 2; OSIVQ -1= Object imagery; OSIVQ -2= Visual imagery; OSIVQ -3= Verbal imagery; E= Extraversion; N=Neuroticism; PNQ= Preference for nature; CNS= Connectedness to Nature; PANAS, P =Positive and Negative Affective Status, positive emotions; PANAS, N =Positive and Negative Affective Status, negative emotions; FDS= Forward Digit span; BDS=Backward Digit span; NML= Number of Missing Landmarks; SQRTCO= SQuare Root of the Canonical Organization (accuracy considering the NML); CA= Canonical Accuracy (accuracy not considering the NML); PRS-1= Being Away; PRS-2= Fascination; PRS-3= Coherence.

^ score-gains derived from post-test” minus “pretest”

**Table S3.** Repeated ANOVA results’ for the pre- and post-exposure measures of affective status, and memory.

|  | Pre-exposure | | Post-exposure | | *F* | *p* | *n^2^p* |
| --- | --- | --- | --- | --- | --- | --- | --- |
|  | *M* | *SD* | *M* | *SD* |  |  |  |
| *Affective status* |  |  |  |  |  |  |  |
| PANAS-P | 32.725 | 4.889 | 33.063 | 6.599 | 0.228 | 0.634 | 0.003 |
| PANAS-N | 13.425 | 3.299 | 11.050 | 1.713 | 51.164 | < .001 | 0.393 |
| *Memory* |  |  |  |  |  |  |  |
| FDS | 4.162 | 0.947 | 4.438 | 1.077 | 9.968 | 0.002 | 0.112 |
| BDS | 4.050 | 1.113 | 4.237 | 1.150 | 2.578 | 0.112 | 0.032 |

*Note.* PANAS, P =Positive and Negative Affective Status, positive emotions; PANAS, N =Positive and Negative Affective Status, negative emotions; FDS= Forward Digit span; BDS=Backward Digit span

The tolerance and variance inflation factor (VIF) were examined to identify any multicollinearity violation. The tolerance ranged from .571 to .894, and the VIF from 1.119 to 1.788, showing that the assumption of no multicollinearity was met in the full model (Neter et al., 1996).

**Table S4.** Linear regression models on perceived restorativeness scores (total score, and subscales: being away, fascination, and coherence) with: age and gender, connectedness to nature and preference for nature, personality, and visuospatial preferences.

|  | *Total PRS* | | | *PRS-1* | | | *PRS-2* | | | *PRS-3* | | |
| --- | --- | --- | --- | --- | --- | --- | --- | --- | --- | --- | --- | --- |
|  | *β* | *CI* | *p* | *β* | *CI* | *p* | *β* | *CI* | *p* | *β* | *CI* | *p* |
| **Age** | -.206 | [-.48; .01] | .062 | -.187 | [-.18; .02] | .096 | -.294 | [-.14; -.02] | .012 | .077 | [-.07; .14] | .512 |
| Gender | .122 | [-4.06; 10.74] | .371 | .176 | [-1.14; 5.14] | .209 | -.076 | [-2.54; 1.46] | .592 | .213 | [-.92; 5.85] | .151 |
| PNQ | .128 | [-.13; .45] | .284 | .158 | [-.05; .23] | .184 | .213 | [-.01; .17] | .081 | .221 | [-.02; .28] | .082 |
| CNS | .249 | [.03; .67] | .034 | .170 | [-.04; .21] | .166 | .195 | [-.02; .14] | .118 | -.029 | [-.15; .12] | .824 |
| E | .216 | [.01; 2.32] | .048 | .195 | [-.05; .93] | .080 | .105 | [-.17; .46] | .353 | .056 | [-.40; .66] | .633 |
| N | -.072 | [-1.10; .56] | .518 | .071 | [-.24; .46] | .532 | -.156 | [-.38; .07] | .184 | -.230 | [-.75; .02] | .060 |
| OSIVQ -1 | .137 | [-.17; .74] | .216 | .137 | [-.08; .31] | .228 | .129 | [-.05; .19] | .265 | .098 | [-.12; .29] | .414 |
| OSIVQ -2 | -.129 | [-.38; .13] | .341 | .018 | [-.10; .12] | .898 | -.155 | [-.11; .03] | .272 | -.020 | [-.13; .11] | .890 |
| OSIVQ -3 | -.045 | [-.33; .22] | .683 | -.063 | [-.11; .08] | .578 | -.126 | [-.12; .03] | .275 | .002 | [-.12; .13] | .989 |
| Model fit |  | F_(9,79)_=3.0228 |  |  | F_(9,79)_=2.527 |  | F_(9,79)_=2.099 | | | F_(9,79)_=1.420 | | |
| *p* |  | .004 |  |  | .014 |  | .041 | | | .196 | | |
| R^2^ |  | .280 |  |  | .245 |  | .212 | | | .154 | | |

*Note.* PRS-1= Being Away; PRS-2= Fascination; PRS-3= Coherence; OSIVQ -1= Object imagery; OSIVQ -2= Visual imagery; OSIVQ -3= verbal imagery; E= Extroversion; N=Neuroticism; PNQ= Preference for nature; CNS= Connectedness to Nature.

^a^Gender was a dichotomous variable (1=female, 2=male)

**Table S5.** Multiple regression models on benefits of affective status (PANAS positive and negative emotions) and memory (FDS and BDS) with: age and gender, connectedness to nature and preference for nature, personality, and visuospatial preferences.

|  | *PANAS-P* | | | *PANAS-N* | | | *FDS* | | | *BDS* | | |
| --- | --- | --- | --- | --- | --- | --- | --- | --- | --- | --- | --- | --- |
|  | *β* | *CI* | *p* | *β* | *CI* | *p* | *β* | *CI* | *p* | *β* | *CI* | *p* |
| Age | -.167 | [-.22; .04] | .174 | .018 | [-.06; .07] | .883 | .180 | [-.01; .03] | .138 | -.108 | [-.03; .01] | .363 |
| Gender | -.193 | [-6.99; 1.55] | .209 | .080 | [-1.45; 2.51] | .594 | -.215 | [-.89; .15] | .156 | -.358 | [-1.52; -.15] | .018 |
| CNS | .166 | [-.06; .28] | .214 | -.232 | [-.15; .01] | .081 | -.108 | [-.03; .01] | .411 | .136 | [-.01; .04] | .295 |
| PNC | .028 | [-.17; .21] | .829 | .249 | [-.01; .17] | .055 | .022 | [-.02; .03] | .864 | .058 | [-.02; .04] | .643 |
| E | -.031 | [-.75; .58] | .798 | .129 | [-.14; .48] | .280 | -.019 | [-.09; .08] | .874 | -.043 | [-.13; .09] | .715 |
| N | .071 | [-.34; .62] | .572 | -.027 | [-.25; .20] | .826 | -.046 | [-.07; .05] | .710 | .141 | [-.03; .12] | .249 |
| OSIVQ -1 | .183 | [-.07; .46] | .143 | -.184 | [-.21; .03] | .135 | -.075 | [-.04; .02] | .539 | -.068 | [-.05; .03] | .571 |
| OSIVQ -2 | -.049 | [-.17; .12] | .748 | .034 | [-.06; .08] | .819 | -.041 | [-.02; .02] | .781 | -.208 | [-.04; .01] | .159 |
| OSIVQ -3 | -.003 | [-.16; .16] | .982 | .049 | [-.06; .09] | .685 | .048 | [-.02; .02] | .694 | -.181 | [-.05; .01] | .134 |
| Model fit |  | F_(9,79)_=0.786 |  |  | F_(9,79)_=1.011 |  | F_(9,79)_=1.018 | | | F_(9,79)_=1.317 | | |
| *p* |  | .630 |  |  | .440 |  | .435 | | | .244 | | |
| R^2^ |  | .092 |  |  | .115 |  | .116 | | | .145 | | |

*Note.* PRS-1= Being Away; PRS-2= Fascination; PRS-3= Coherence; OSIVQ -1= Object imagery; OSIVQ -2= Visual imagery; OSIVQ -3= verbal imagery; E= Extroversion; N=Neuroticism; PNQ= Preference for nature; CNS= Connectedness to Nature.

^a^Gender was a dichotomous variable (1=female, 2=male)

**Table S6.** Multiple regression models on landmark positioning accuracy (NML, SQRTCO, and CA) with: age and gender, connectedness to nature and preference for nature, personality, and visuospatial preferences.

|  | *NML* | | | *SQRTCO* | | | *CA* | | |
| --- | --- | --- | --- | --- | --- | --- | --- | --- | --- |
|  | *β* | *CI* | *p* | *β* | *CI* | *p* | *β* | *CI* | *p* |
| Age | -.025 | [-.05; .04] | .831 | .029 | [-.01; .01] | .810 | .027 | [-.0; .01] | .823 |
| Gender | .153 | [-.64; 2.03] | .303 | -.110 | [-.13; .06] | .468 | .069 | [-.06; .10] | .653 |
| PNC | .023 | [-.05; .06] | .860 | -0.096 | [-.01; .01] | .459 | -.244 | [-.01; .01] | .068 |
| CNS | -.193 | [-.10; .01] | .130 | 0.234 | [-.01; .01] | .068 | .211 | [-.01; .01] | .105 |
| E | -.001 | [-.21; .21] | .991 | 0.060 | [-.01; .02] | .613 | .182 | [-.01; .02] | .134 |
| N | -.111 | [-.22; .08] | .362 | 0.067 | [-.01; .01] | .591 | .063 | [-.01; .01] | .616 |
| OSIVQ -1 | -.165 | [-.14; .03] | .173 | .207 | [-8.12; .01] | .091 | -.181 | [-.01; .02] | .146 |
| OSIVQ -2 | -.151 | [-.07; .02] | .305 | .150 | [-.01; .01] | .314 | .222 | [-.01; .01] | .144 |
| OSIVQ -3 | .126 | [-.02; .08] | .297 | -.108 | [-.01; .01] | .368 | .042 | [-.01; .01] | .733 |
| Model fit |  | F_(9,79)_=1.296 |  |  | F_(9,79)_=1.393 |  | F_(9,79)_=1.091 | | |
| *p* |  | .255 |  |  | .209 |  | .382 | | |
| R^2^ |  | .143 |  |  | .158 |  | .128 | | |

*Note.* PRS-1= Being Away; PRS-2= Fascination; PRS-3= Coherence; OSIVQ -1= Object imagery; OSIVQ -2= Visual imagery; OSIVQ -3= verbal imagery; E= Extroversion; N=Neuroticism; PNQ= Preference for nature; CNS= Connectedness to Nature.

^a^Gender was a dichotomous variable (1=female, 2=male)

**
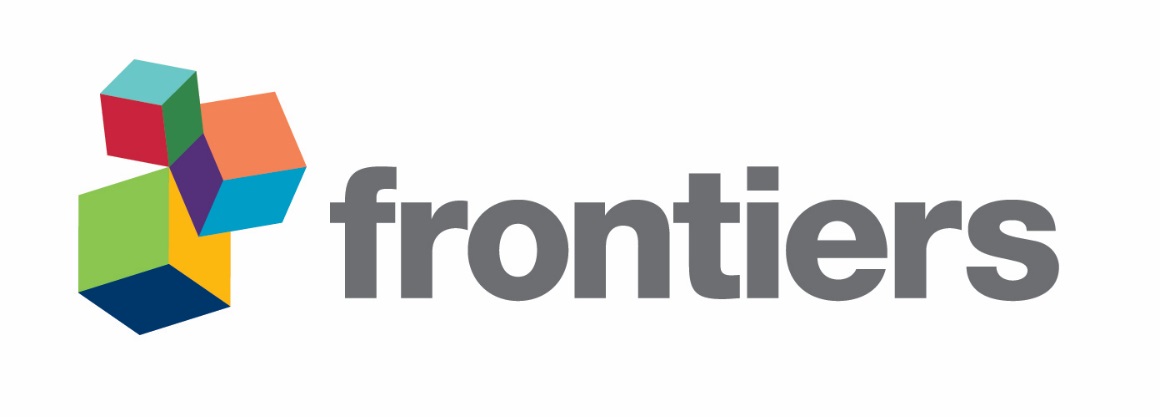
**
